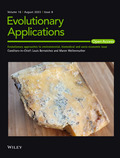# Cover Image

**DOI:** 10.1111/eva.13407

**Published:** 2023-08-23

## Abstract

Caption: A slice of a French Termignon blue cheese naturally colonized by *Penicillium roqueforti*.

Credit: Tatiana Giraud.